# PALB2: research reaching to clinical outcomes for women with breast cancer

**DOI:** 10.1186/s13053-016-0049-2

**Published:** 2016-04-19

**Authors:** Melissa C. Southey, Ingrid Winship, Tú Nguyen-Dumont

**Affiliations:** Genetic Epidemiology Laboratory, Department of Pathology, The University of Melbourne, Parkville, VIC 3010 Australia; Department of Medicine, The University of Melbourne, Parkville, VIC 3010 Australia; The Royal Melbourne Hospital, Parkville, VIC 3050 Australia

**Keywords:** *PALB2*, Breast cancer susceptibility, Cancer susceptibility, Familial cancer

## Abstract

*PALB2* has taken its place with *bona fide* breast cancer susceptibility genes. It is now well established that women who carry loss-of-function mutations in the *PALB2* gene are at similarly elevated breast cancer risks to those who carry mutations in *BRCA2*. Information about *PALB2* is now being used in breast cancer clinical genetics practice and is routinely included in breast cancer predisposition gene panel tests. Tens of thousands of women worldwide have now had genetic tests for *PALB2* mutations in the context of breast cancer susceptibility. However, prospective data related to the clinical outcomes of *PALB2* mutation carriers is lacking and very little information (beyond mutation penetrance) is available to guide current clinical management for carriers (affected and unaffected by cancer). In addition, clinical classification of the vast array of non-loss-of-function genetic variants identified in *PALB2* is in its infancy. These are key areas of current research efforts and are important foundations on which to move information about *PALB2* into the precision public health arena.

## Introduction

For the last two decades, women have been offered genetic testing of *BRCA1* and *BRCA2* in various clinical contexts. The vast majority of these women are seeking an explanation for a personal or family history of breast and/or ovarian cancer, and an accurate means of risk assessment, to facilitate risk management across the family. Indeed clinical criteria used to determine eligibility for *BRCA1* and *BRCA2* testing in many settings have been founded on the number of affected relatives and their age at diagnosis and then developed over time with increased evidence and local practice issues. Of those women who undergo testing, up to 20 % are found to carry a clinically actionable mutation in *BRCA1* or *BRCA2*. Until very recently additional genetic testing was not possible unless other clinical indicators were present (such as Li-Fraumeni syndrome that might indicate genetic testing of *TP53*). Women and their families who received uninformative genetic test results for *BRCA1* and *BRCA2* were clinically managed solely on the basis of their personal and family history. This limited the use of invasive strategies such as risk reduction surgery.

Continued research and a recent revolution in genetic technology that can be applied to this research has identified a number of additional breast cancer predisposition genes and reported a large number of additional candidate breast cancer predisposition genes that are yet to be validated. This same technology has also transitioned into molecular diagnostic laboratories and has enabled a shift from high cost single gene genetic tests to lower cost multi-gene panel tests. The uptake of gene panel tests has been rapid and included a volume of successful direct-to-the-public marketing. In some areas of clinical genetics, panel testing is now the standard of care [1]. With some important caveats and considerations discussed in this review, current data suggests that gene panel testing offers breast cancer clinical genetics practice increased opportunity to identify “actionable” genetic variants in a greater proportion of women [2–4].

However, few of the large number of genes included in many gene panel tests are *bona fide* breast cancer predisposition genes and the vast majority of genetic variation identified by these gene panel tests cannot be interpreted in terms of breast or ovarian cancer risk. This is currently a controversial area of breast cancer research and clinical genetics practice, and is discussed in detail elsewhere [5].

*PALB2* has now firmly taken its place with the small number of *bona fide* breast cancer susceptibility genes. It is now well established that women who carry mutations in the *PALB2* gene are at similar breast cancer risks as those who carry mutations in *BRCA2* [6, 7]) making many rethink the appropriateness of the initial “moderate or intermediate risk gene” label [8]. *PALB2* now plays a legitimate role in breast cancer clinical genetics practice and takes a valid place on breast cancer predisposition gene panel tests. Internationally, tens of thousands of women, including those who have gone direct to the test provider, have had genetic tests for *PALB2* mutations in the context of breast cancer susceptibility. Today, many nations have (or are preparing) best practice guidelines that include recommendations for *PALB2* genetic testing and risk management [5, 9].

Currently, prospective data related to the clinical outcomes of *PALB2* mutation carriers is lacking and very little information (beyond mutation penetrance) is available to guide clinical management for carriers (affected and unaffected by breast cancer). Over the last two decades, evidence has slowly been accumulated to support recommendations around risk management and targeted treatment regimes for *BRCA1* and *BRCA2* mutation carriers. Very little of this evidence currently exists for *PALB2* mutation carriers.

Accumulating this evidence is challenging due to the very low frequency of women with *PALB2* mutations, even in affected women with a strong family history of breast and ovarian cancer. However, with new technology and international coordination there is promise that further evidence could be gathered for *PALB2* mutation carriers that will improve their clinical care within a few years.

In addition, risk estimates for *PALB2* mutations have been based on collections of loss-of-function mutations. Clinical classification of the vast array of non-loss-of-function genetic variants identified in *PALB2* is in its infancy. Informed by prior research in this area involving unclassified genetic variants in *BRCA1* and *BRCA2,* international initiatives are moving quickly to identify the best approaches to assess *PALB2* genetic variants on a variant-by-variant basis, to enable personalized use in clinical genetics practice.

*PALB2* has made it over the first hurdle and is now included in the breast cancer clinical genetics arena but to extend current utility and have an impact on improving the clinical outcomes for carriers of *PALB2* mutations and incorporate use of this genetic information into precision public health initiatives, additional data is still urgently required.

## *PALB2: A bona fide* breast cancer susceptibility gene

Mutations in *PALB2* make a small contribution to heritable breast cancer susceptibility in most populations. Germline *PALB2* mutations and carrier frequencies have been reviewed elsewhere [10]. Briefly, protein truncating mutations in *PALB2* are distributed throughout the coding region [6, 10] yet four *PALB2* mutations are of note in terms of multiple observations: *PALB2* c.509_510delGA (p.Arg170fs*14) in Poland [11], *PALB2* c.2323C > T (p.Gln775*) in French Canadians [12], *PALB2* c.1592delT (p.531 fs*30) in Finland [13] and *PALB2* c.3113G > A identified in affected women in the UK, USA and Australia [8, 14–17]. Reports from a variety of populations consistently estimate that *PALB2* mutations are associated with increased risk for breast cancer (e.g., OR 3.94, 95 % CI, 1.5–12.1) [13].

As few studies have been conducted within unselected breast cancer cases, estimation of the age-specific cumulative risk (penetrance) of breast cancer associated with *PALB2* mutations has been limited. The family histories of the case carriers, unselected for age or family history, reported in Northern Finland [13], were used to estimate *PALB2* c.1592delT to be associated with a 40 % (95 % CI, 17 %–77 %) risk of breast cancer to the age of 70 years [18]. Similarly, an Australian population-based case–control-family study of breast cancer estimated the cumulative risk for *PALB2* c.3113G > A to be 91 % (95 % CI, 44–100 %) to the age of 70 years [17].

To consider penetrance of a larger number of *PALB2* genetic variants and a larger number of families, the PALB2 interest group [19] embarked on a collaborative effort that collected data from 362 members of 154 families who had deleterious truncating, splice, or deletion mutations in *PALB2* [6]. The estimated average cumulative risk of breast cancer risk ranged from 33 % (95 % CI, 25–44 %) for a female carrier without affected relatives to 58 % (95 % CI, 50–66 %) for a female carrier with two first-degree relatives who had breast cancer diagnosed by 50 years of age. Supported by other similar observations [20], some recommend that both family history and *PALB2* genotype should be considered together for clinical breast cancer risk management.

Thus, all published estimates of penetrance of *PALB2* mutations are comparable to the breast cancer risk associated with *BRCA2* mutations: 45 % (95 % CI, 31–56 %) [7]. *PALB2* is now regarded as a *bona fide* breast cancer predisposition gene and is justifiably included on current breast cancer gene testing panels with the above evidence.

## Evidence-based translation into breast cancer clinical genetics practice

**Fig. 1:**
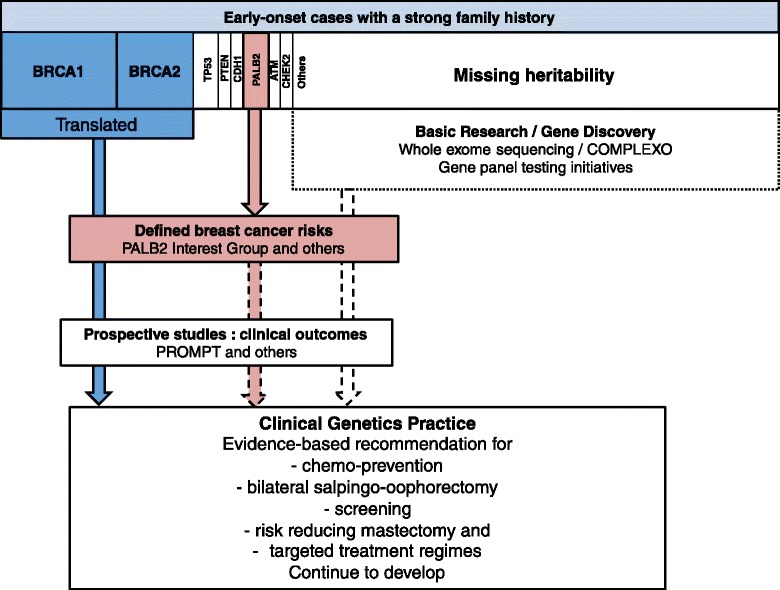
PALB2: evidence-based translation into practice. Based on the Australian Breast Cancer Family Registry and the Australian context

An overview of the pathway to translation of new genetic information related to breast cancer clinical genetics practice is shown in Fig. 1. This figure represents women diagnosed with breast cancer under the age of 40 years with a strong family history of breast and or ovarian cancer (two or more effected first or second degree relatives). Pathogenic mutations in *BRCA1* and *BRCA2* have been identified in approximately 25 % of these women. The risk associated with carrying these mutations and the clinical outcomes for these women (collectively) have been well characterised and this evidence is used to inform the clinical management of these women and their families [21]. Loss-of-function *PALB2* mutations are identified in approximately 2 % of these women [17]. Breast cancer risk associated with carrying a *PALB2* loss-of-function mutations is now established but research is urgently needed to extend this knowledge to an understanding of clinical outcomes for carriers. Rare mutations in genes associated with genetic syndromes such as Li-Fraumeni (*TP53*), Cowden syndrome (*PTEN*) and hereditary diffuse gastric cancer (*CDH1*) are also present in this group of women at very low frequency. Specific evidence related to clinical outcomes for breast cancer in these contexts is lacking. Additional breast cancer susceptibility genes including *ATM* and *CHEK2* also require prospective data to provide the evidence-base for clinical decision making around risk management/reduction and treatment options. Several other genes have been identified as candidate breast cancer predisposition genes that require further validation such as *FANCM* [22] and *RECQL* [23]. The remaining early-onset breast cancer cases with a strong family history remain the subject of research trying to identify the “missing heritability” of breast cancer via numerous initiatives including COMPLEXO [24].

## *PALB2* mutations and risk of other cancer types

As PALB2 functions together with BRCA1 and BRCA2, in the same DNA-damage response pathway, it has been thought plausible that *PALB2* mutations, similar to *BRCA1* and *BRCA2* mutations, could predispose to other cancer types. The rarity of mutations in *PALB2* and the rarity of some of the other cancers likely to be involved (pancreatic, male breast cancer, ovarian cancer, prostate cancer) make the estimation of the risk (if any) extremely challenging. Data in this area continues to come from small (yet important) studies and case reports that accumulatively may assist this interpretation ([25] and many others). By pooling international resources, the PALB2 Interest Group estimated that the relative risk of ovarian cancer and male breast cancer for *PALB2* mutation carriers was 2.31 (95 % CI, 0.77–6.97; *P* = 0.18) and 8.30 (95 % CI, 0.77–88.56; *P* = 0.08) respectively [6].

*PALB2* germline mutations are rare in cases of pancreatic cancer [26–32]. A recent report describing the mutational landscape of pancreatic cancers has illustrated the potentially important role for paralleling somatic and germline mutation testing to enable the identification not just of the rare germline *PALB2* mutation carriers but also the mutation signature of DNA damage repair deficiency in pancreatic cancers that could benefit from platinum therapies [33].

Some substantial opportunities to assess risk for other cancers have come from application of targeted sequencing projects and gene panel testing. In the context of risk for ovarian cancer, Ramus et al. have reported sequencing results for *PALB2* for 3,236 women affected with epithelial ovarian cancer and 3,431 unaffected women. Nine mutation carriers were identified in the affected women and three in the unaffected women (*P* = 0.08). Interestingly they also identified seven carriers in 2,000 unaffected women with a family history of breast or ovarian cancer (all women had at least 10 % lifetime risk) (*P* = 0.045 when compared to the controls above) [34]. Norquist et al. have reported findings from a targeted capture and massively parallel sequencing assay that included the identification of 12 *PALB2* mutation carriers in a group of 1,915 women with ovarian cancer unselected for family history (*P* = <0.001 when compared to data from the Exome Sequencing Project and the Exome Aggregation Consortium) [35]. Two protein truncating mutations in *PALB2* (c.1592delT, p.Leu531Cysfs and c.3113G > A, p.Trp1038*) were included on the iCOGS and genotyped in 16,287 ovarian cancer cases and 23,491 controls. No evidence for association with ovarian cancer risk was observed (OR 2.50, 95 % CI, 0.21–29.1), *p* = 0.45 and OR 1.34, 95 % CI, 0.36–4.97), *p* = 0.66 respectively) (Southey MC, Goldgar D, Winqvist R, Pylkas K, Couch FJ, Tischkowitz M.: PALB2, CHEK2 and ATM rare variants and cancer risk: data from COGS, unpublished).

There is still very little data and no evidence supporting an association between *PALB2* mutations and prostate cancer risk [13, 36–39] although several pedigrees have been presented and a possible trend toward aggressive disease in carriers has been noted [39]. iCOGS measured *PALB2* (c.1592delT, p.Leu531Cysfs and c.3113G > A, p.Trp1038*) in 22,301 prostate cancer cases and 22,320 controls and found no evidence for association with prostate cancer risk OR 2.06, 95 % CI 0.59–7.11, *p* = 0.24 and OR 0.49, 95 % CI, 0.18–1.36, *p* = 0.16 respectively.

The *PALB2* interest group continues work to further refine breast and other cancer risks for *PALB2* mutation carriers [19].

## PALB2 mutation carriers: clinical outcomes

As described above, recent work has increased the precision of breast cancer risk estimates for *PALB2* mutation carriers providing some new information with clinical utility. However, prospective data related to the clinical outcomes of *PALB2* mutation carriers is lacking and very little information (beyond mutation penetrance) is available to guide current clinical management for carriers (affected and unaffected by breast cancer).

It has taken decades of research to provide the evidence base for *BRCA1* and *BRCA2* mutation carriers to make informed decision about the use of chemo-preventive agents, the use of the bilateral salpingo-oophorectomy, the use of mammography, magnetic resonance imaging (MRI) and other screening modalities, risk reducing mastectomy and targeted treatment regimes. Accumulating this evidence was challenging due in part to the very low frequency of women with *BRCA1 and BRCA2* mutations, the historically laborious and expensive process of testing for mutations in these genes and the need to follow these women prospectively.

However, in today’s context where *PALB2* is being included in gene panel tests that are being conducted rapidly in large numbers at reduced costs and research can be conducted in a coordinated fashion internationally involving well established research resources (including resources founded to assess these questions for *BRCA1* and *BRCA2* mutation carriers) and in community-academic-industry partnerships – there is promise that evidence can be found for *PALB2* mutation carriers that will impact clinical practice in the short term.

In the only report of its kind so far, Cybulski et al*.,* identified 116 carriers of either 509_510delGA or 172_175delTTGT in 12,529 women diagnosed with invasive breast cancer in Poland. Ten-year survival for women with breast cancer and a *PALB2* mutation was 48.0 % (95 % CI, 36.5 %–63.2 %), compared with 74.7 % (95 % CI, 73.5 %–75.8 %) for non-carriers (hazard ratio for death 2.27, 95 % CI, 1.64–3.15; *p* < 0.0001) [40]. Some other data indirectly supports a likely worse outcome for *PALB2* mutation carriers, including the propensity in some population for *PALB2* mutation-associated breast cancers to be of higher grade, including triple negative. However, these reports are few and small in size, are variable in findings and suggest *PALB2* mutation-specific tumour phenotypes [14, 15, 20, 41].

Further studies are required to test if women who carry *PALB2* mutations are at increased risk of death from breast cancer compared to non-carriers. More information is needed to understand the options for prevention and risk reduction. Intuitively, given the similar biological role of the protein, it is likely that some of the recommendations for *BRCA1* and *BRCA2* mutation carriers, including therapeutic regimes, may be relevant for *PALB2* mutation carriers – but much work is needed to resolve these questions.

To this end, a new academic-industry partnership named PROMPT – **P**rospective **R**egistry **o**f **M**ulti**p**lex **T**esting [42, 43], and many other large research initiatives are underway to address these important questions for carriers. PROMPT has scope beyond addressing these questions for *PALB2* alone and will support the rapid translation of similar information for several new breast cancer predisposition genes including *ATM*, *CDH1*, *CHEK2*, *RAD51C*, *RAD51D*, *STK11*, *TP53* in addition to *BRCA1* and *BRCA2*. PROMPT is an online research registry for people who have undergone gene panel testing and been found to have a genetic variation in one of the above genes. PROMPT is one of several initiatives that create a new paradigm for research study participation that directly involves the most relevant community. PROMPT is designed to involve those who want to share their genetic results, learn more from sharing these results and engage at a level of their choosing/comfort as a collaborator alongside physicians and researchers to learn more about how mutations in these genes (such as *PALB2*) may affect their health and cancer risks.

## Classification of rare variants

In contrast to several other breast cancer predisposition genes, there is no evidence that missense variants in *PALB2* (as a combined group) are associated with increased risk for breast cancer [44, 45]. We and others have reported that the breast cancer risk fraction contributed by missense variants in *BRCA1*, *BRCA2*, *ATM* and *CHEK2* is as high, if not higher, than protein-truncating variants in these genes [46–48]. However, interpretation of the rare genetic variation observed in *PALB2* on a variant-by-variant basis, especially the rare missense variants, remains challenging [44]. That is, on a variant-by-variant basis it is difficult provide any information that can be used to guide clinical management of carriers of rare missense mutations.

In some practices, the previous approach of breast cancer clinical genetics to generalize risk within groups of similar mutations (e.g., protein truncating mutations in *BRCA1*) has not been automatically applied in the context of *PALB2* due to i) a perception that the *PALB2* risk estimates currently include data from a disproportionate number of the founder *PALB2* mutations (and thus may not represent the average risk associated with all loss-of-function mutations) and ii) the more recent characterisation of variants in *BRCA1* (e.g., R1699Q [49, 50]) and *BRCA2* (e.g., K3326* [51, 52]) with more moderate or low risk of breast cancer that is also a plausible scenario for variants in other genes, including *PALB2*.

There is therefore a need to extend international efforts that are currently trying to classify rare variants identified in *BRCA1* and *BRCA2* for clinical use to include rare variants identified in *PALB2* (and other genes) to assist the clinical management of the individuals who carry them. Several activities are well underway.

The most extensive and internationally set groups working in this area include The PALB2 Interest Group [6, 19] and The Evidence-based Network for the Interpretation of Germline Mutation Alleles (ENIGMA) [53–55] whose members are providing a range of data to accumulate new evidence on a variant-by-variant basis to be assessed in multifactorial risk models. These groups are also providing expert opinion to global databases and classification initiatives and working to communicate new information to clinical genetics practices urgently in need of individualized information.

The assessment and clinical classification of rare missense variants in *PALB2* are likely to require incorporation of many pieces of evidence to enable clinical utility. Some of this evidence is likely to come from so-called functional assays. Fortunately, several functional domains of PALB2 are recognized including a coiled-coil structure, an ETGE-type KEAP1 binding motif, a chromatin-association motif (ChAM) at the N-terminus and a WD repeat motif in the C-terminus (reviewed elsewhere [10]). These domains, coupled with PALB2’s role in DNA repair and Fanconi anemia, are facilitating work that is pitched at assessing the functional differences between wildtype PALB2 and PALB2 carrying rare missense mutations in key functional domains. Park et al., characterized effects of missense mutations of the PALB2 WD40 domain and demonstrated that PALB2 L939W (c.2816 T > G) and PALB2 L1143P (c.3428 T > A) display a decreased capacity for DNA double-strand break-induced homologous recombination and an increased cellular sensitivity to ionizing radiation [56]. This data offers much potentially useful information for rare variant classification - yet key questions still need to be answered. For instance, 1) how should these assays be calibrated (i.e., how much change of function equates to increased cancer risk)?; 2) is the magnitude of functional change proportional to magnitude of cancer risk?; 3) how should these results be coded, weighted and incorporated into multifactorial risk models?

Calibrated assays for functional assessment of variants in *BRCA1* and *BRCA2* have been developed and reported [57, 58]. Recently, a publically available resource for functional analysis of missense variants in *BRCA1* (*BRCA1* Circos) has been made available to facilitate meta-analysis of functional data and improve classification of variants in that gene [59]. It is anticipated that groups such as the Functional Working Group of ENIGMA [60] will be able to develop similar resources for PALB2 once assays are further developed and data is available.

## Conclusions: towards precision medicine

There is much well founded optimism in the research community that a number of large initiates (some detailed here) will generate data quickly, on the necessary scale and pooled to make definitive analyses related to variant classification and clinical outcomes for *PALB2* carriers a reality in the near future.
